# CXCL1 induces senescence of cancer-associated fibroblasts via autocrine loops in oral squamous cell carcinoma

**DOI:** 10.1371/journal.pone.0188847

**Published:** 2018-01-23

**Authors:** Eun Kyoung Kim, Sook Moon, Do Kyeong Kim, Xianglan Zhang, Jin Kim

**Affiliations:** 1 Oral Cancer Research Institute, Department of Oral Pathology, Yonsei University College of Dentistry, Seoul, Republic of Korea; 2 Department of Dental hygiene, College of nursing Healthcare, Sorabol college, Gyeongju, Republic of Korea; 3 Department of pathology, Yanbian University Hospital, Yanji City, Jilin Province, China; University of Bergen, NORWAY

## Abstract

Cancer-associated fibroblasts (CAFs) have emerged as one of the main factors related to cancer progression, however, the conversion mechanism of normal fibroblasts (NOFs) to CAFs has not been well elucidated. The aim of this study was to investigate the underlying mechanism of CAF transformation from NOFs in oral squamous cell carcinoma (OSCC). This study found that NOFs exposed to OSCC cells transformed to senescent cells. The cytokine antibody array showed the highest secretion levels of IL-6 and CXCL1 in NOFs co-cultured with OSCC cells. Despite that both IL-6 and CXCL1 induced the senescent phenotype of CAFs, CXCL1 secretion showed a cancer-specific response to transform NOFs into CAFs in OSCC, whereas IL-6 secretion was eventuated by common co-culture condition. Further, CXCL1 was released from NOFs co-cultured with OSCC cells, however, CXCL1 was undetectable in mono-cultured NOFs or co-cultured OSCC cells with NOFs. Taken together, this study demonstrates that CXCL1 can transform NOFs into senescent CAFs via an autocrine mechanism. These data might contribute to further understanding of CAFs and to development of a potential therapeutic approach targeting cancer cells-CAFs interactions.

## Introduction

Molecular interactions between cancer cells and their surrounding stroma have a crucial role in carcinogenesis [1,2]. Fibroblasts supporting cancer cells, the most common type of stromal cells, are termed cancer-associated fibroblasts (CAFs) or tumor-associated fibroblasts [3,4]. We adopted the term of CAFs in this study. The important functions of CAFs include the deposition of extracellular matrix (ECM), regulation of epithelial differentiation, and cancer initiation and progression [2,5].

CAFs are considered activated myofibroblasts with expression of alpha-smooth muscle actin (α-SMA)[6]. These myofibroblastic CAFs potently promote the proliferation of cancer cells [7,8]. A recent study demonstrated that the myofibroblastic characteristics of CAFs are mediated by transforming growth factor-β (TGF-β) and stromal cell-derived factor-1 in mammary fibroblasts of breast cancer[9]. Although not all CAFs express α-SMA, CAFs are considered activated fibroblasts, as evidenced by increased expression of proinflammatory genes, such as chemokine (C-X-C motif) ligand 1 (CXCL1, Gro-α), CXCL2 (Gro-β), interleukin 1β (IL-1β), and interleukin 6 (IL-6) [10].

On the other hand, accumulating data have demonstrated that CAFs are regarded as senescent cells and contribute to cancer progression in various human cancers [11–13]. Senescent cells actively communicate with their microenvironment through cytokines secretion named as senescence-associated secretory phenotype. With secreted cytokines, senescent fibroblasts stimulate the growth of preneoplastic and malignant epithelial cells but not of normal epithelial cells [14]. In addition, senescent fibroblasts stimulate the migration and invasion of immortalized or premalignant epithelial cells [15,16]. Whatever defined as activated or senescent phenotypes of CAFs, it is apparent that CAFs activated by cancer cells express proinflammatory cytokines, promoting cancer progression.

In light of the importance of the role of CAFs in carcinogenesis, the investigation of transformation mechanism to CAFs may contribute to the further understanding of interaction between cancer cells and CAFs and the development of new therapeutic targets in human cancer. The aim of this study was to investigate the conversion mechanism of normal fibroblasts (NOFs) into CAFs in oral squamous cell carcinoma (OSCC). This study demonstrates that CXCL1 plays a role in transformation of NOFs into CAFs via an autocrine manner.

## Materials and methods

### Cell isolation and cell cultures

CAFs were obtained from the surgical specimens of 3 OSCC patients, whose ages were 54, 69 and 74 years old, respectively. NOFs were derived from 3 patients who underwent wisdom tooth extraction without mucosal disease. All of 3 patients were 29 years old. This work has been carried out in accordance with the Declaration of Helsinki and the informed consent was received from the patients. These procedures were approved by the Institutional Review Board (IRB) of Yonsei University College of Dentistry (IRB 2-2012-0027). NOFs and CAFs were then maintained in culture medium composed of Dulbecco’s modified Eagles medium (Gibco BRL, NY, USA) and F-12 Ham (Gibco BRL, NY, USA) mixed in a 3:1 ratio, and supplemented with 10% fetal bovine serum and 1% penicillin/streptomycin. The 5^th^ ~ 9^th^ passaged cells were used for this study. Normal human epidermal keratinocytes (NEK) and two types of OSCC cells (YD10B, YD38) were also used for this study[17,18]. The details were described in the supplementary materials and methods (S1 Materials and Methods).

### Treatments of recombinant proteins, control antibody and neutralizing antibody

The recombinant human proteins, IgG_2B_ isotype control antibody, and CXCL1 neutralizing antibody were purchased from R&D Systems, Minneapolis, MN, USA. To evaluate α-SMA protein expression in NOFs and CAFs, recombinant human TGF-β1 protein (10 ng/ml) was treated for 48 h. To examine senescent effects of IL-6 and CXCL1, we first carried out preliminary experiments to check the concentration of each cytokine secreted in mono-cultured or co-cultured NOFs with OSCC cells for 48 h (S1 Table). Based on these data, the concentration of recombinant human IL-6 (7 ng/ml) and CXCL1 (5 ng/ml) were applied in NOFs for 48 h. The concentration of CXCL1 neutralizing antibody (20 μg/ml) was determined by preliminary data (S1 Table). IgG_2B_ isotype control antibody (20 μg/ml) was also used in transwell invasion assay. Treatments of recombinant human proteins, IgG_2B_ isotype control antibody, and CXCL1 neutralizing antibody were conducted in serum-free culture conditions.

### Reverse transcription–polymerase chain reaction (RT-PCR) and real-time PCR

Total RNA was extracted from each cell using an RNeasy plus mini kit (Qiagen, Hilden, Germany), and complementary DNA was synthesized using 2.5 × RT-&GO^TM^ Mastermix (MP Biomedicals, Santa Ana, CA, USA) according to the manufacturer’s instructions. Primer sequences for RT-PCR and real-time PCR are presented in Table 1. In RT-PCR, complementary DNA was amplified by using Accu Power Hot Start PCR Pre Mix (Bioneer, Daejeon, South Korea) with conditions of 28 ~ 33 cycles of 30 s at 94°C, 40 s at 55 ~ 60°C, and 40 s at 72°C. The amplified products were separated on 1.5% agarose gel stained with 0.1 μg/ml of ethidium bromide, and photographed under UV light (Bio-Rad, Hercules, CA, USA). Real-time PCR was carried out using the SYBR Green I Master (Roche Applied Science, Mannheim, Germany) and normalized to GAPDH. The result was analyzed by using the LightCycler 480 software (Roche Applied Science, Mannheim, Germany).

**Table 1:** Primer sequences used for RT-PCR and Real-time PCR.

Genes	Sense (5' →3')	Antisense (5' →3')
α-SMA	GGCCGAGATCTCACTGACTA	AGTGGCCATCTCATTTTCAA
FAP	ACTGCCCAGTTCGTTTCAGT	AGAGCGACCCTCACATCAAG
Vimentin	GACAATGCGTCTCTGGCACGTCTT	TCCTCCGCCTCCTGCAGGTTCTT
Groα/CXCL1	TGTGAAGGCAGGGGAATGTA	TTAAGCCCCTTTGTTCTAAGCC
GAPDH	GAAGGTGAAGGTCGGAGT	GAAGATGGTGATGGGATTTC

### Western blots

Cell lysates were separated by SDS-polyacrylamide gel (Bio-Rad, Hercules, CA, USA), transferred onto a polyvinylidene fluoride membrane (Bio-Rad, Hercules, CA, USA), and then exposed to the appropriate antibodies: α-SMA (1:100, mouse monoclonal, Dako, Glostrup, Denmark), and β-actin (1:1000, rabbit polyclonal, Sigma, St. Louis, MO, USA). The membranes were incubated with horseradish peroxidase-conjugated anti-mouse or rabbit antibodies (1:2000, Cell Signaling, Beverly, MA, USA) and were visualized by chemiluminescence (GenDEPOT, Barker, TX, USA).

### Immunocytochemistry

NOFs and CAFs (1.5 × 10^5^) were placed onto 22-mm glass coverslips (Deckglaser, Germany) for 24 h before staining. The cells were fixed with 95% ethanol for 20 min at room temperature. Endogenous peroxidase activities were inactivated in 3% hydrogen peroxide solution for 10 min at room temperature, and the cells were blocked with 5% bovine serum albumin (Sigma, St. Louis, MO, USA) for 30 min at room temperature. The cells were immunostained with anti-proliferating cell nuclear antigen (anti-PCNA, 1:100; Dako, Glostrup, Denmark) at room temperature for 1 h. Peroxidase-labeled anti-mouse/rabbit IgG (Dako, Glostrup, Denmark) was then applied at room temperature for 30 min. The cells were visualized with 3,3-diaminobenzidine tetrachloride (Dako, Glostrup, Denmark) and counter-stained with Mayer’s hematoxylin. Images of 5 random microscopic fields (magnification: X200) were acquired per sample by a light microscopy (Olympus, Tokyo, Japan). The percentage of PCNA-positive cells was calculated by dividing the number of positive cells by the total number of cells. Immunofluorescence staining for α-SMA expression in NOFs and CAFs was described in the supplementary materials and methods (S2 Materials and Methods).

### Senescence-associated β-galactosidase (SA-β-Gal) staining

In brief, NOFs and CAFs (1.5 × 10^5^) were seeded and incubated overnight in a 6-well plate. After treatment of IL-6, CXCL1 or co-culture with OSCC cells for 48 h, cells were stained using a β-Gal staining kit (Cell Signaling, MA, USA) and incubated overnight at 37°C. The cells were washed with PBS twice, and 70% glycerol was added. Images of randomly selected 5 microscopic fields (magnification: X200) were acquired per sample (Olympus, Tokyo, Japan). The percentage of SA-β-Gal-positive cells was calculated by the number of positive cells dividing by the total number of cells.

### Frozen cancer tissues samples from OSCC patients

To confirm the *in vitro* data, SA-β-Gal staining was also performed with frozen surgical cancer tissues obtained from 5 OSCC patients, and their tumor-free marginal tissues were used as controls (IRB approval number: 2-2011-0044). This work has been carried out in accordance with the Declaration of Helsinki and the informed consent was received from the patients. Cryocut-sectioned tissue was stained using a β-Gal staining kit (Cell Signaling, MA, USA) and counter-stained with eosin. All frozen tissue sections contained both tumor and stromal portions. The stromal proportion was measured by cellSens standard software (ROI; region of interest) (Olympus, Australia) in randomly selected 5 fields per slide, and then the stained cells were counted. The SA-β-Gal-positive cells were calculated per millimeter squared and averaged for each group.

### Cytokine antibody array

A RayBio® Human Cytokine Antibody Array kit (Ray Biotech, Norcross, GA, USA) was used to screen cytokine secretion in conditioned medium of each culture. The cytokine antibody array was performed according to the manufacturer’s protocol. In brief, mono-cultured NOFs and co-culture NOFs with YD10B cells were seeded in 6-well plates. After 48 h incubation, the conditioned mediums were collected, concentrated and quantified for array. The detail of conditioned medium was described in the supplementary materials and methods (S3 Materials and Methods). Among 80 cytokines, the relative expression levels of the cytokines were determined by comparing signal intensities. Image J software (National Institutes of Health, Bethesda, Maryland, USA) was used for densitometric analysis.

### Enzyme-linked immunosorbent assay (ELISA)

ELISA was performed according to the manufacturer’s protocol (R&D systems, Minneapolis, MN, USA). Preparation of conditioned medium was described in the supplementary data. All reagents for ELISA were purchased from R&D Systems. Capture antibodies were applied in 8-well NUNC Immuno Modules (Thermo Fisher Scientific, Roskilde, Denmark) and incubated overnight at 4°C. Wells were blocked with 1% bovine serum albumin (Sigma, St. Louis, MO, USA) at room temperature for 1 h. The conditioned medium was applied at room temperature for 2 h. Then, detection antibodies were applied at room temperature for 1 h. One hundred μl of 3,3‘,5,5‘-tetramethylbenzidine (TMB) solution (Sigma, St. Louis, MO, USA) was added and color reaction was stopped by adding 50 μl of 2M sulfuric acid. The optical density was measured at 450 nm in a micro-plate reader (Bio-Rad, Hercules, CA, USA). To measure each secretory cytokine, a standard curve with a range of 0 ~ 2000 pg/ml was prepared by making serial dilution of recombinant proteins.

### Measurement of oxidative stress

Reactive oxygen species (ROS) were measured with the fluorescent probe 2’7’-dichlorofluorescin diacetate (H_2_DCFDA) dye (Molecular Probes Inc) according to the instructions. The cells (2.5 × 10^5^) were exposed to the 10 μM H_2_DCFDA in PBS in the dark at 37°C for 20 min, respectively. H_2_DCFDA-non treatment served as a negative control and 10 μM H_2_O_2_ served as a positive control. Then, the cells were analyzed by a flow cytometry (Becton Dickinson, Beckman coulter, Fullerton, CA, USA) at an excitation and emission wavelength of 485 nm and 535 nm, respectively. Cell-Quest software (BD Biosciences, San Jose, CA, USA) was utilized for data analysis.

### Transwell invasion assay

Inserts containing 8-μm pores in 24-transwell plates (Corning incorporated-Life Sciences, Tewksbury, MA, USA) were coated with type I collagen (Nitta Gelatin Inc, Osaka, Japan) (45 μg/30 μl/well) and hardened for 24 h. OSCC cells (1.5 × 10^4^) were placed in the inserts coated with type I collagen. NOFs or CAFs (1.5 × 10^4^) were added to the lower chamber of the well. A CXCL1-neutralizing antibody was added to evaluate whether CXCL1 induced the invasive growth of cancer cells. After 48 h, the penetrated cells through the pores of inserts were fixed, stained with 0.25% crystal violet and counted by light microscopy (Olympus, Tokyo, Japan).

### Statistical analysis

All statistical analyses were performed using SPSS version 20 (SPSS Inc., Chicago, IL, USA). Mann-Whitney *U* tests were used to assess the significance of mRNA and protein expressions between NOFs and CAFs. Mann-Whitney *U* tests were also used to assess the significance of SA-β-Gal enzyme activity, ROS generation, and cytokine secretion levels between mono-culture and co-culture conditions. An analysis of variance (ANOVA) with repeated measurements was used to assess the significance of proliferation and SA-β-Gal enzyme activity between NOFs and CAFs depending on the passages. Donor age served as a co-variable to exclude age effect in the repeated measures ANOVA. The results were reported as the mean ± standard deviation (SD). A value of *p* < 0.05 was considered statistically significant.

## Results

### Comparison of the fraction of proliferating and senescent cells between NOFs and CAFs

**Fig 1:**
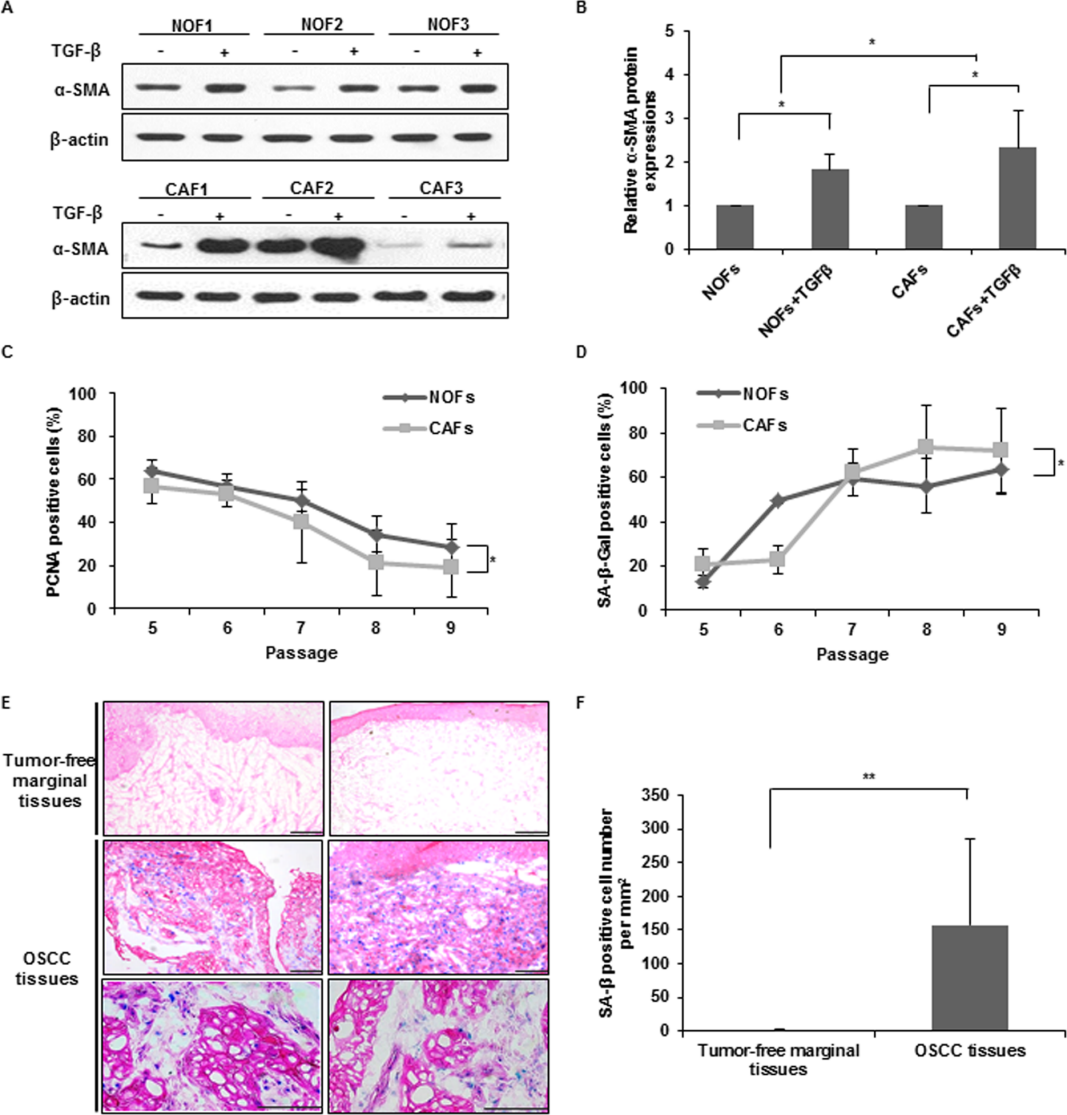
Comparison between NOFs and CAFs. (A, B) α-SMA protein expression according to TGF-β treatment in NOFs and CAFs. β-actin expression was used as a loading control. (B) Densitometric analysis of α-SMA protein expression in NOFs and CAFs with normalization to β-actin level. The results are presented as the mean value ± SD in triplicates and were analyzed by the Mann-Whitney *U* test (*p < 0.05). (C) The percentage of PCNA-positive cells in NOFs (n = 3) and CAFs (n = 3). Images of randomly selected 5 microscopic fields (magnification: X200) per sample were selected and then calculated. (D) The percentage of SA-β-Gal-positive cells in NOFs (n = 3) and CAFs (n = 3). Images of randomly selected 5 microscopic fields (magnification: X200) per sample were selected and then calculated. The results of C and D are presented as the mean value ± SD and were analyzed by the repeated measures ANOVA (*p < 0.05). (E) Representative microscopic pictures of SA-β-Gal-positive cells in frozen surgical OSCC specimens and its tumor-free marginal tissues (magnification: X200 and X400, scale bar: 100 μm). (F) The average number of SA-β-Gal-positive cells per square millimeter in frozen surgical OSCC specimens and its tumor-free marginal tissues. The results are presented as the mean value ± SD and were analyzed by the Mann-Whitney *U* test (*p < 0.05, **p < 0.01).

First, we examined the expression of myofibroblastic markers. The mRNA expression of α-SMA and FAP increased in both TGF-β-treated NOFs and CAFs compared with non-treated cells, whereas vimentin mRNA expression was not altered (S1A–S1E Fig). The basal level of α-SMA expression showed no statistical difference between NOFs and CAFs. Upon TGF-β treatment in NOFs and CAFs, the expression difference of α-SMA protein exhibited statistical significance between CAFs and NOFs (*p < 0.05) (Fig 1A and 1B, S2 Fig). To compare the proliferation between NOFs and CAFs, immunocytochemical staining for PCNA was performed (S3A Fig) and analyzed by the repeated measures ANOVA using a co-variable (Fig 1C, S2 Table). Regarding the big age gap of between NOF (all 29 years) and CAF (54, 69 and 74 years) donors, their age served as a co-variable in statistical analysis to exclude age effect. Donor age served as a co-variable in statistical analysis to exclude age effect. CAFs exhibited a significantly reduced fraction of PCNA positive cells compared with NOFs overall passage over from 5^th^ to 9^th^ passage (*p < 0.05). To detect senescent cells, SA-β-Gal staining was performed (S3B Fig) and analyzed using the same statistical method (Fig 1D, S3 Table). CAFs exhibited a significantly higher fraction of SA-β-Gal-positive cells compared with NOFs overall passage over from 5^th^ to 9^th^ passage (*p < 0.05). Confirming these results, the stromal area surrounding carcinoma cells in OSCC patients exhibited a higher fraction of SA-β-Gal-positive cells than the area in tumor-free marginal tissues samples (**p < 0.01) (Fig 1E and 1F).

### Senescence of NOFs by exposure to cancer cells

**Fig 2:**
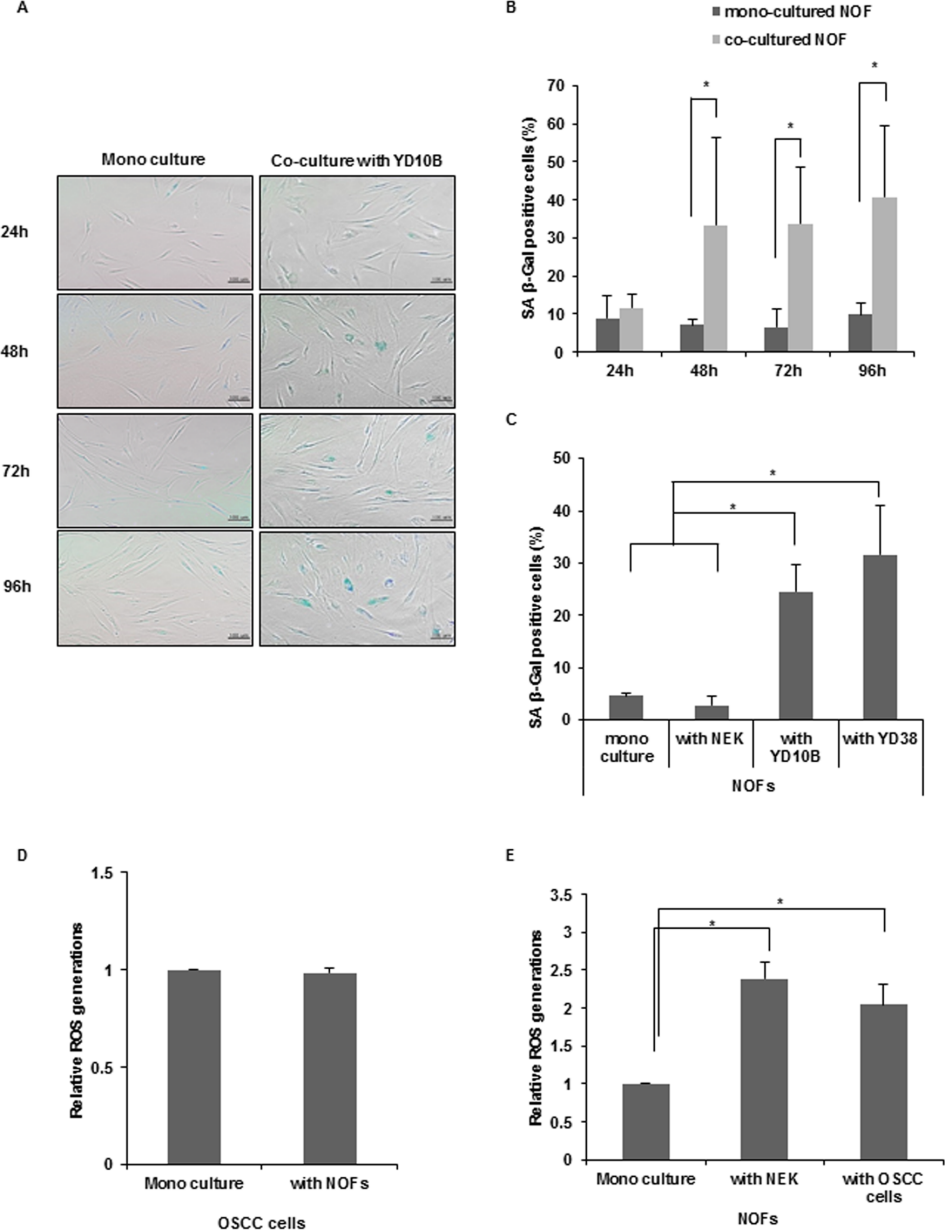
Comparison of senescent NOFs between mono-culture and co-culture. (A) Representative microscopic pictures of SA-β-Gal positive cells at 24, 48, 72, and 96 h (magnification: 200X, scale bar: 100 μm). (B) The SA-β-Gal positive cells were normalized by dividing the total cells and are presented as % of SA-β-Gal positive cells. The results are presented as the mean value ± SD in triplicates and were analyzed by the Mann-Whitney *U* test (*p < 0.05). (C) The number of SA-β-Gal-positive cells among co-cultures with NEK, OSCC cells at 48 h. The results are presented as the mean value ± SD in triplicates and were analyzed by the Mann-Whitney *U* test (*p < 0.05). (D, E) Measurement of oxidative stress in mono-culture and co-culture conditions. Flow cytometry analysis of positive cell stained H_2_DCFDA dye for detection of ROS generation. (D) Quantitative analysis of ROS generation in mono-cultured and co-cultured OSCC cells with NOFs. The results are presented as the mean value ± SD in triplicates and were analyzed by the Mann-Whitney *U* test. (E) Quantitative analysis of ROS generation in mono-cultured and co-cultured NOFs with OSCC cells or NEK. The results are shown as mean value ± SD in triplicates and were analyzed by the Mann-Whitney *U* test (*p < 0.05).

To investigate whether NOFs transform to senescent CAFs by exposure to cancer cells, SA-β-Gal staining was performed after co-culture with NOFs and YD10B OSCC cells at 24, 48, 72 and 96 h time points (Fig 2A and 2B). At 48 h, NOFs co-cultured with YD10B exhibited significantly increased SA-β-Gal-positive cells compared with mono-cultured NOFs (*p < 0.05). To confirm the transformation of NOFs into senescent CAFs, the percentage of SA-β-Gal-positive cells was compared at 48 h. NOFs co-cultured with OSCC cells exhibited a significantly high percentage of SA-β-Gal-positive cells (*p < 0.05), whereas NOFs co-cultured with NEK exhibited no positive cells, suggesting that senescence of NOFs is attributed to the exposure to cancer cells (Fig 2C).

### ROS generation in mono-culture and co-culture conditions

To evaluate whether senescence of NOFs by exposure to cancer cells is due to ROS generation in co-culture condition, we measured ROS. No significant difference in ROS generation was observed between mono-cultured and co-cultured OSCC cells with NOFs (Fig 2D and S5 Fig). Co-cultured NOFs showed higher ROS generation than mono-cultured cells, but no significant difference was observed between NOFs co-cultured with NEK and OSCC cells (Fig 2E and S5 Fig).

### Cytokine profiles of NOFs in co-culture with OSCC cells

**Fig 3:**
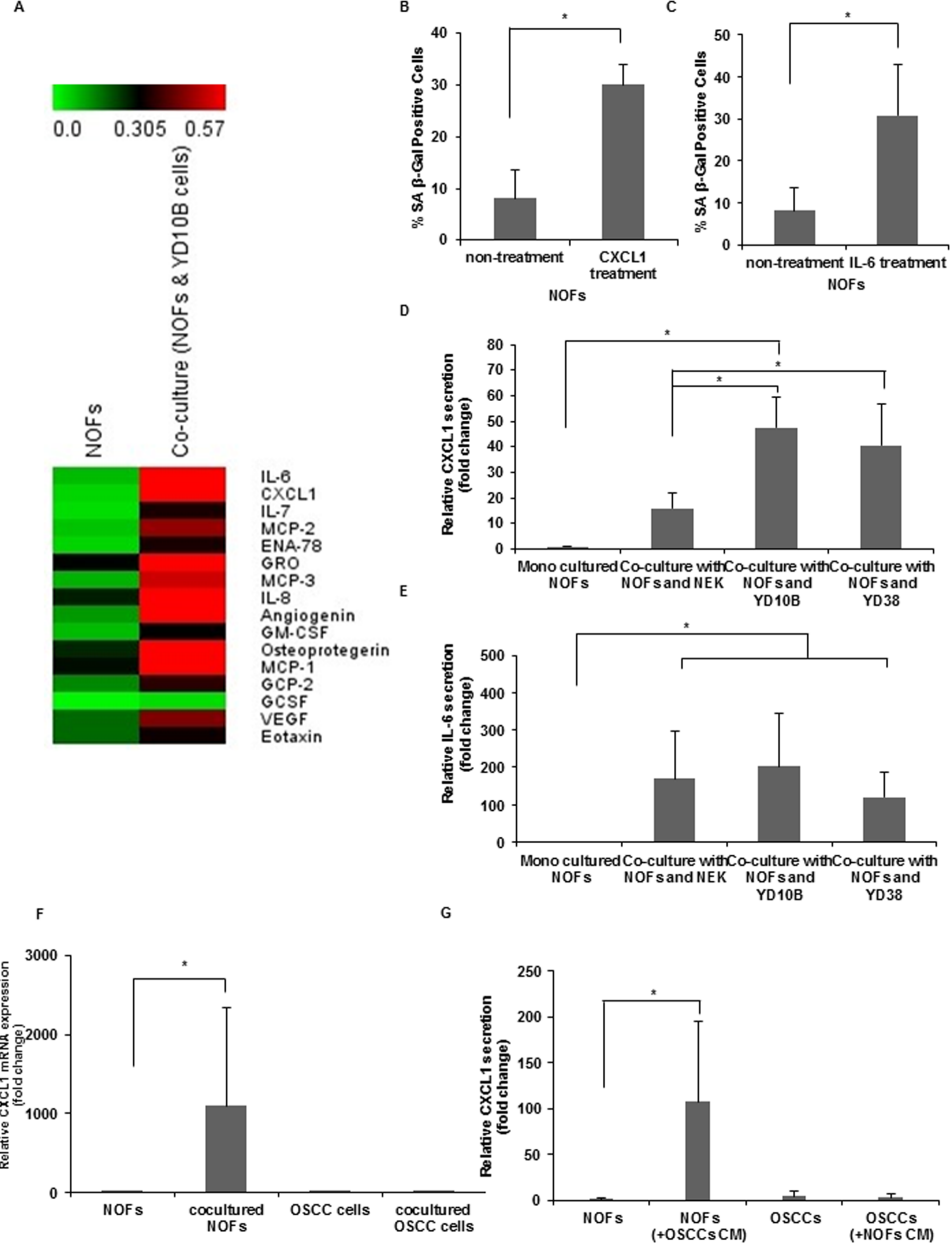
CXCL1 secretion by an autocrine manner in CAFs. (A) A cytokine antibody array was used to measure the secretion of 80 factors in conditioned medium from mono-cultured NOFs and co-cultured NOFs with OSCC cells for 48 h. The arrays were scanned and quantified by densitometry using Image J software. The levels were normalized to internal positive controls present in each membrane. The heat map shows upregulated cytokines in co-culture with NOFs and OSCC cells using the MeV 4.9.0 program. (B,C) The number of SA-β-Gal positive cells by CXCL1 (B) and IL-6 treatment (C). The results are presented as the mean value ± SD in triplicates and were analyzed by the Mann-Whitney *U* test (*p < 0.05). (D,E) The amount of secreted CXCL1 (D) or IL-6 (E) was measured by ELISA in conditioned medium (CM) from NOFs, OSCC cells, co-cultures of NOFs and NEK, and co-cultures of NOFs and OSCC cells. CXCL1 secretion was quantified by a standard curve prepared by serial dilution of recombinant proteins. The results are presented as the mean value ± SD in triplicates and were analyzed by the Mann-Whitney *U* test (*p < 0.05). (F,G) CXCL1 expressions were measured by real-time PCR (F) and ELISA (G) in mono-culture NOFs, OSCC cells, NOFs co-cultured with OSCC cells, and OSCC cells co-cultured with NOFs. (F) The mRNA expressions were normalized to GAPDH. (G) The amount of secreted CXCL1 in mono-culture NOFs, OSCC cells, NOFs treated with CM from OSCC cells, and OSCC cells treated with CM from NOFs. The results are presented as the mean value ± SD in triplicates and were analyzed by the Mann-Whitney *U* test (*p < 0.05).

To elucidate the soluble factors to induce senescence of NOF, a cytokine antibody array was performed (S4 Fig). Sixteen cytokines exhibited greater than 1.5-fold increases in co-cultured NOFs with YD10B cells compared with mono-cultured NOFs, of which IL-6 and CXCL1 exhibited the highest secretion levels (Fig 3A). IL-6 and CXCL1 exhibited a 24.25-fold and 19.94-fold increase, respectively (Table 2).

**Table 2:** A Cytokines list over a 1.5-fold increases in co-culture with NOFs and YD10B OSCC cells compared with mono cultured NOFs.

Order	Target	Fold-increase
1	IL-6	24.25
2	CXCL1	19.94
3	IL-7	7.66
4	MCP-2	6.68
5	ENA-78	6.54
6	GRO	5.96
7	MCP-3	5.85
8	IL-8	5.26
9	Angiogenin	4.72
10	GM-CSF	3.71
11	Osteoprotegerin	3.15
12	MCP-1	2.81
13	GCP-2	2.61
14	GCSF	2.51
15	VEGF	2.49
16	Eotaxin	1.75

### Specificity of CXCL1 in co-culture condition with OSCC cells

Based on the cytokine antibody array results, the senescent effects of IL-6 and CXCL1 were examined. Upon treatment with IL-6 or CXCL1 recombinant proteins, a significantly increased percentage of SA-β-Gal-positive cells was detected in treated cells compared with non-treated NOFs (*p < 0.05) (Fig 3B and 3C, S6A and S6B Fig). To evaluate whether these cytokines are released by stimulation of cancer cells or by simple co-culture effect, IL-6 and CXCL1 secretion levels were measured in conditioned medium from mono-culture, co-culture with NEK, and co-culture with OSCC cells. In co-culture conditions, regardless of whether with OSCC cells or NEK, IL-6 and CXCL1 cytokine were highly secreted compared with mono-cultured condition (*p < 0.05) (Fig 3D and 3E). However, CXCL1 level specifically increased in co-culture with NOFs and OSCC cells compared with co-culture with NOFs and NEK (Fig 3D). IL-6 level showed no significant difference between co-culture with OSCC cells and with NEK (Fig 3E). These results indicated that secretion of CXCL1 in CAFs was triggered specifically by cancer cells.

To identify the main source of CXCL1, real-time PCR and ELISA were conducted. CXCL1 mRNA exhibited the highest expression in co-cultured NOFs with OSCC cells (Fig 3F). Likewise, CXCL1 secretion was highly detected in NOFs treated with conditioned medium obtained from OSCC cells (*p < 0.05). In contrast, CXCL1 secretion was minimally detected in mono-cultured NOFs, mono-cultured OSCC cells, and OSCC cells treated with conditioned medium from NOFs (Fig 3G). These results indicated that CXCL1 is specifically secreted when exposed to OSCC cells in an autocrine manner.

### Effects of CXCL1 inhibition in CAF senescence and OSCC invasion

**Fig 4:**
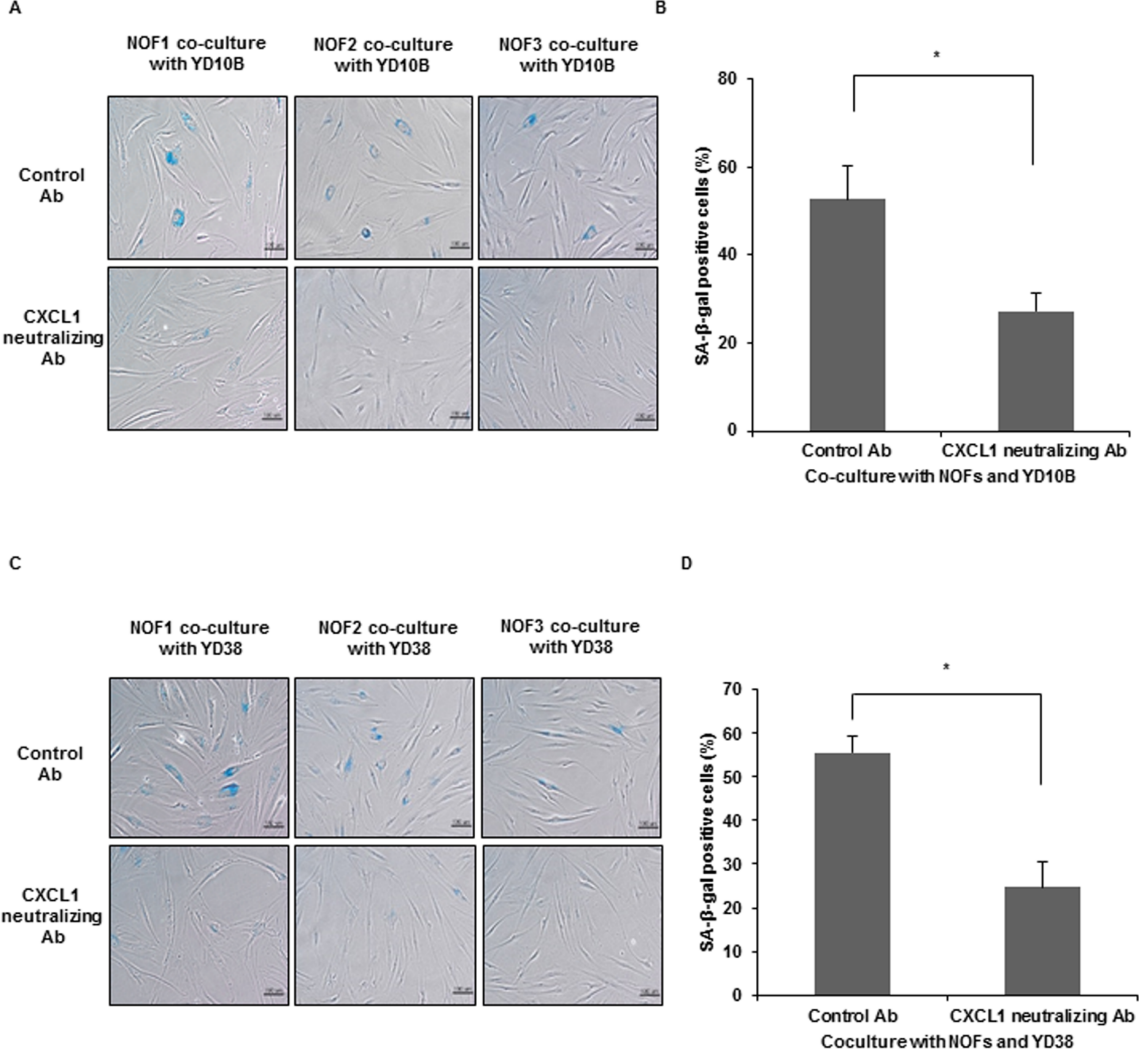
Reduction of SA-β-Gal positive cells by CXCL1-neutralizing antibody. (A,C) Representative microscopic pictures of SA-β-Gal positive cells in co-cultured NOFs with YD10B (A) or YD38 cells (C) after treatment with neutralizing antibody (Ab). (magnification: 200X, Scale bar: 100 μm). (B,D) The percentage of SA-β-Gal-positive cells was normalized by dividing the total cells. The results are presented as the mean value ± SD in triplicates and were analyzed by the Mann-Whitney *U* test (*p < 0.05).

To confirm the induction role of senescence by CXCL1, a CXCL1-neutralizing antibody was treated in co-culture conditions with NOFs and OSCC cells. The number of SA-β-Gal-positive cells was significantly decreased by treatment with CXCL1-neutralizing antibody (*p < 0.05) (Fig 4).

**Fig 5:**
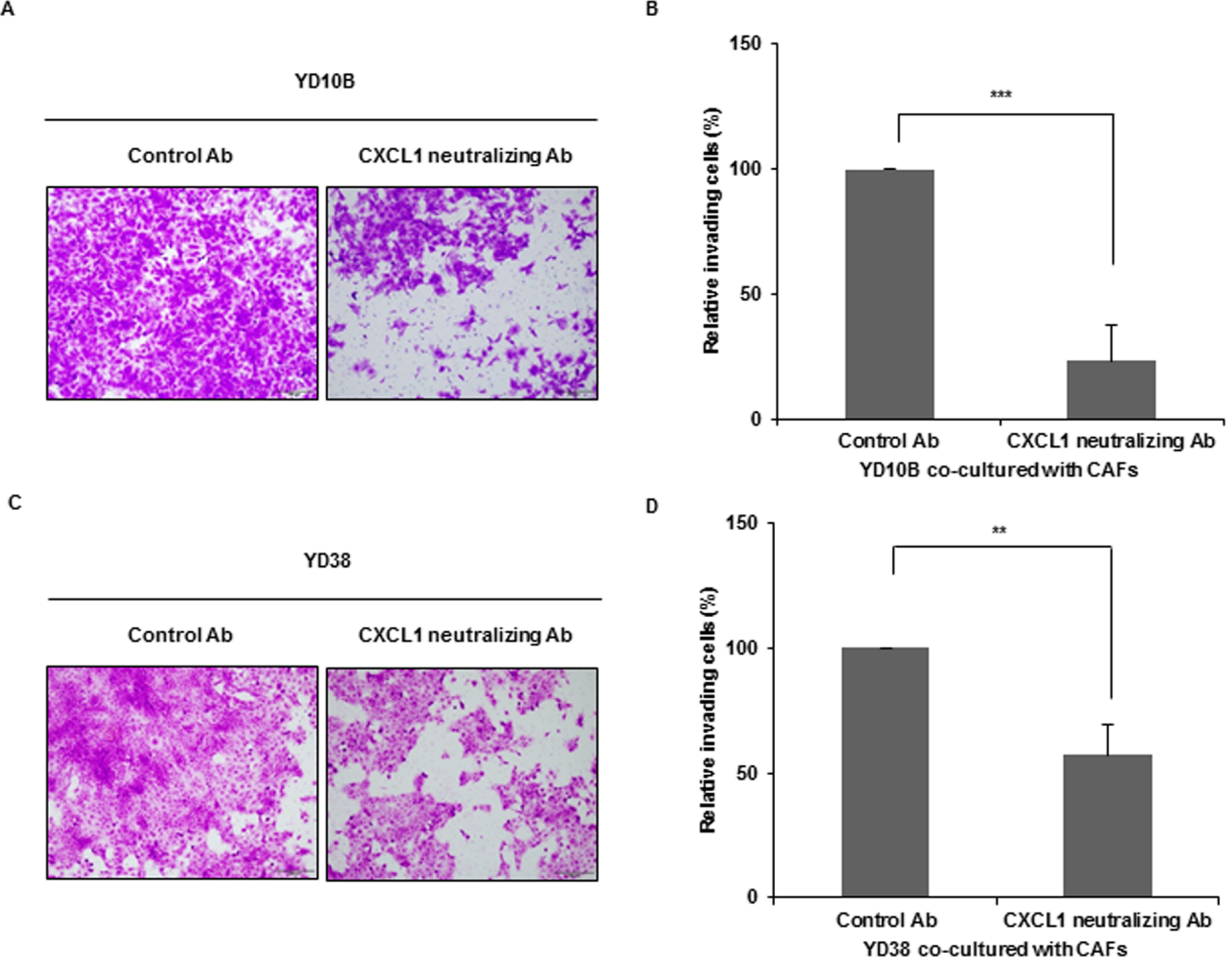
The invasive growth of CXCL1 in CAFs. YD10B (A,B) or YD38 (C,D) cells in serum-free media were placed in the upper well of a 24-transwell plate with collagen-coated filters (8 μm pore). CAFs were added into the lower well. The invasive cells were compared after treatment with control antibody or CXCL1-neutralizing antibody. The invasive cells were counted after 48 h by light microscopy. (A,C) Representative microscopic pictures of invading OSCC cells (magnification: 100X, scale bar: 200 μm). (B,D) Comparison of invasiveness after treatment of CXCL1-neutralizing antibody. The number of invasive cells was normalized by dividing by the number of total cells and presented as the percentage of invasion. The results are presented as the mean value ± SD in triplicates and were analyzed by the Mann-Whitney U test (***p < 0.001, **p < 0.01, *p < 0.05).

To determine whether CAFs are involved in the induction of cancer cell invasion, transwell invasion assays were performed in the presence of NOFs or CAFs in the lower chamber. OSCC cells co-cultured with CAFs exhibited higher invasive activity than co-cultured with NOFs (*p < 0.05, **p < 0.01) (S7 Fig). With CXCL1-neutralizing antibody, the invasiveness of OSCC cells was markedly reduced compared with controls (**p < 0.01, ***p < 0.001) (Fig 5), suggesting that CXCL1 is involved in CAF-induced OSCC cell invasion.

## Discussion

CAFs have various functions in the induction of cancer development and progression [2,19]. However, the conversion mechanism of NOFs into CAFs has not been clearly elucidated to date. This study revealed that NOFs in OSCC are transformed to senescent CAFs mediated by CXCL1 in an autocrine manner.

CAFs have been revealed to be myofibroblasts with α-SMA expression [5,20]. In our study, we could not find statistical difference in the basal level of α-SMA expression between NOFs and CAFs, because of individual variation, supporting the previous data that α-SMA expression are diverse per cell [21]. The individual variation of α-SMA expression appeared greater in CAFs than in NOFs. TGF-β-treated CAFs exhibited significantly increased α-SMA protein expression compared with TGF-β-treated NOFs, confirming myofibroblastic differentiation of CAFs by TGF-β [9]. Accumulating evidence demonstrates that CAFs have a senescent phenotype in several cancers [12,22]. In our *in vitro* results, CAFs showed the higher portion of senescent cells than NOFs overall passage over, confirming that CAFs are senescent cells. Consistent with *in vitro* findings, OSCC patient tissues exhibited an increased fraction of SA-β-Gal-positive cells compared with tumor-free marginal tissues, confirming that CAFs are senescent cells.

According to our results, upon co-culturing NOFs with OSCC cells for 48 h, NOFs can be transformed into senescent cells. To confirm whether this phenomenon was a specific response to co-culture between NOFs and OSCC cells, SA-β-Gal positivity of NOFs was compared in co-culture with NEK and with OSCC cells. SA-β-Gal-positive cells were not detectable in NOFs co-cultured with NEK, indicating that senescent process of NOFs may not be attributed to simply co-culture condition, but be attributed to exposure by cancer cells. Taken together, NOFs co-cultured with OSCC cells *in vitro* for 48 h can be educated to CAFs.

Considering that ROS play a major role in aging process and senescent CAFs [23], we measured ROS generation among mono-cultured and co-cultured conditions. Co-cultured fibroblasts with NEK or OSCC cells showed higher ROS generation than mono-cultured cells, but not significant difference in co-culture between with NEK and with OSCC cells, suggesting that senescent change of NOFs to CAFs by exposure to cancer cells may not be attributed to ROS.

In our study, the proliferating activity of CAFs, as shown in Fig 1C and 1D, rapidly reduced over the passages compared with NOFs, eventuating in arrested proliferation rate. In contrast, the senescence of CAFs rapidly increased compared with NOFs. According to our previous study [5], the higher portion of CAFs caused the poorer prognosis. We also showed that CAFs can proliferate approximately 1.5 fold by stimulation of OSCC cells, lending support that CAFs proliferation can aggravate cancer progression by inducing the larger amount of cytokine secretion from the more number of CAFs. Regarding that cytokine secretion of senescent CAFs has been acknowledged to play a critical role in cancer progression, we focused on elucidating the transforming mechanism of NOFs to senescence cells.

We found that IL-6 and CXCL1 showed the highest secretory factors by co-culture of NOFs and OSCC cells. IL-6 is a multifunctional cytokine that regulates cell proliferation, survival, senescence, and differentiation and promotes tumorigenesis [24]. In addition, IL-6 is a molecular modulator of cellular adjustment to an altered environment [25]. CXCL1 contributes to cancer cell transformation, growth and invasion [26,27]. In our data, we found that both IL-6 and CXCL1 induced senescence of CAFs. However, CXCL1 secretion appeared to be cancer specific, whereas IL-6 did not.

Accumulating data demonstrated that the control of senescence in CAFs may give an insight to develop a new cancer therapy targeting tumor microenvironment. In cervical tumorigenesis, the activated IL-6 and STAT-3 induced senescence of fibroblasts in high-risk Human Papilloma Virus-infected cervical cancer. The suppression of STAT-3 pathway reduced tumor burden, suggesting the possibility of cancer therapy targeting senescence program of CAFs [12]. As another possibility of cancer therapy, NF-κB acts as a master regulator of senescence associated secretory phenotype. The controls of NF-κB pathway contributes to the outcome of cancer therapy [28].

CXCL1 was derived from NOFs by exposure to OSCC cells, suggesting that senescent process of CAFs occurs in an autocrine manner in our study model. The previous studies demonstrated that CAFs-derived CXCL1 can be a therapeutic target in cancer therapy. In ovarian cancer, RAS-induced CXCL1 can be a potent inducer of senescence in stromal fibroblasts, showing the possibility of diagnostic marker and therapeutic target of CXCL1 [11,29]. Regarding an analogy in paracrine interactions between epithelial and stromal fibroblasts in both wound healing and carcinogenesis, the blockage of IL-6, IL-8 and CXCL1 released from stromal fibroblasts may attenuate epithelial differentiation [11,29]. In addition, CAFs-derived CXCL1 mediated radioresistance in esophageal squamous cell carcinoma, providing that CXCL1 can be an attractive target to reverse tumor radioresistance [30]. In our study, the treatment of CXCL1-neutralizing antibody significantly reduced invasive growth in OSCC cells, supporting the usefulness of target therapy for CAFs-derived CXCL1. Taken together, these data might contribute to the further understanding of CAFs and to the development of a therapeutic target modulating cancer cells and CAFs interactions.

## Supporting information

S1 Fig Comparison of myofibroblastic markers between NOFs and CAFs. (A,B) α-SMA, FAP, and vimentin mRNA expression according to TGF-β treatment in NOFs (A) and CAFs (B). The graphs are presented as the mean value ± SD in triplicates. (C, D, E) Densitometric analysis of mRNA for α-SMA (C), FAP (D), and vimentin (E) were carried out with a loading control of GAPDH expression. The results are presented as the mean value ± SD in triplicates and were analyzed by the Mann-Whitney *U* test (*p < 0.05).
(TIF)

S2 Fig Representative immunofluorescent pictures of α-SMA in NOFs and CAFs. Images are shown the basal levels of α-SMA in NOFs(upper) and CAFs(lower). Merged staining was shown (DAPI(blue), α-SMA(Green)). (magnification: X200, scale bar: 100μm).
(TIF)

S3 Fig Representative microscopic pictures of PCNA and SA-β-Gal staining in NOFs and CAFs. (A) The positive cells for PCNA show the brown to black colored nuclei. (B) The positive cells for SA-β-Gal show the blue colored cytoplasm and nuclei. (magnification: X 200, scale bar: 100μm).
(TIF)

S4 Fig Cytokine antibody array using mono-cultured NOFs and co-cultured NOFs with YD10B OSCC cells. (A) The RayBio Human Cytokine Antibody Array Map. A total of 80 antibodies against cytokines, negative control (Neg), and positive control (Pos) were included in the array (B) Representative pictures of cytokine antibody array in mono-culture NOFs and co-cultured NOFs with YD10B OSCC cells. IL-6 (identified by red empty squares) and CXCL1 (identified by blue empty squares) are the highest secretion in conditioned medium from co-culture with NOFs and YD10B OSCC cells compared to mono-cultured cells.
(TIF)

S5 Fig Measurement of oxidative stress in mono-culture and co-culture conditions. Flow cytometry analysis of positive cell stained H_2_DCFDA dye for detection of ROS generation in mono-culture and co-culture condition. (A) Negative (H_2_DCFD-non treatment) and positive (10 μM H_2_O_2_ treatment) control (B) mono-cultured OSCC cells and OSCC cells co-cultured with NOFs (C, D, E) mono-cultured NOFs and NOFs co-cultured with OSCC cells.
(TIF)

S6 Fig Representative microscopic pictures of SA-β-Gal positive cells in NOFs treated with recombinant proteins. The treatment with CXCL1 (A) and IL-6 recombinant protein (B) (magnification: 200X, Scale bar: 100 μm).
(TIF)

S7 Fig Comparison of invasiveness between NOFs and CAFs by transwell assay. YD10B (A,B) or YD38 (C,D) cells in serum-free media were placed in the upper well of a 24-transwell plate with collagen-coated filters (8 μm pore). NOFs or CAFs was added into the lower well to induce invasion. The invasive cells were counted after 48 h by light microscopy. (A,C) Representative microscopic pictures of invading YD10B or YD38 OSCC cells (magnification: 100X, scale bar: 100 μm). (B,D) The number of invasive cells was normalized by dividing by the number of total cells and presented as the percentage of invasion. The results are presented as the mean value ± SD in triplicates and were analyzed by the Mann-Whitney U test (**p < 0.01, *p < 0.05).
(TIF)

S1 Table The preliminary study for optimal concentration of IL-6, CXCL1 and CXCL1 neutralizing antibody. The preliminary tables indicated to check the concentration of each cytokine secreted in mono-cultured or co-cultured NOFs with OSCC cells for 48 h. For following experiments, the optimal concentration of recombinant human IL-6 (7 ng/ml; Top table) and CXCL1 (5 ng/ml; Middle table) were applied in NOFs for 48 h. The optimal concentration of CXCL1 neutralizing antibody (20 μg/ml; bottom table) was determined as the most effective reduction of CXCL1 secretion.
(DOCX)

S2 Table The percentage of PCNA-positive cells in NOFs and CAFs according to passages. Images of randomly selected 5 microscopic fields (magnification: X200) were acquired per sample (Olympus, Tokyo, Japan). The average (%) was indicated with standard deviation.
(DOCX)

S3 Table The percentage of SA-β-Gal-positive cells in NOFs and CAFs according to passages. Images of randomly selected 5 microscopic fields (magnification: X200) were acquired per sample (Olympus, Tokyo, Japan). The average (%) was indicated with standard deviation.
(DOCX)

S1 Materials and Methods Cell culture. (DOCX)

S2 Materials and Methods Immunofluorescence. (DOCX)

S3 Materials and Methods Preparation of conditioned medium. (DOCX)

## References

[1] Tlsty TD, Coussens LM. Tumor stroma and regulation of cancer development. Annu Rev Pathol. 2006;1: 119–150. doi: 10.1146/annurev.pathol.1.110304.100224 18039110 10.1146/annurev.pathol.1.110304.100224

[2] Kalluri R, Zeisberg M. Fibroblasts in cancer. Nat Rev Cancer. 2006;6: 392–401. doi: 10.1038/nrc1877 16572188 10.1038/nrc1877

[3] Orimo A, Gupta PB, Sgroi DC, Arenzana-Seisdedos F, Delaunay T, Naeem R, et al Stromal fibroblasts present in invasive human breast carcinomas promote tumor growth and angiogenesis through elevated SDF-1/CXCL12 secretion. Cell. 2005;121: 335–348. doi: 10.1016/j.cell.2005.02.034 15882617 10.1016/j.cell.2005.02.034

[4] Kunz-Schughart LA, Knuechel R. Tumor-associated fibroblasts (part I): Active stromal participants in tumor development and progression? Histol Histopathol. 2002;17: 599–621. doi: 10.14670/HH-17.599 11962761 10.14670/HH-17.599

[5] Bae JY, Kim EK, Yang DH, Zhang X, Park YJ, Lee DY, et al Reciprocal interaction between carcinoma-associated fibroblasts and squamous carcinoma cells through interleukin-1alpha induces cancer progression. Neoplasia. 2014;16: 928–938. doi: 10.1016/j.neo.2014.09.003 25425967 10.1016/j.neo.2014.09.003 PMC4240921

[6] Mueller MM, Fusenig NE. Friends or foes—bipolar effects of the tumour stroma in cancer. Nat Rev Cancer. 2004;4: 839–849. doi: 10.1038/nrc1477 15516957 10.1038/nrc1477

[7] Olumi AF, Grossfeld GD, Hayward SW, Carroll PR, Tlsty TD, Cunha GR. Carcinoma-associated fibroblasts direct tumor progression of initiated human prostatic epithelium. Cancer Res. 1999;59: 5002–5011. 10519415 10.1186/bcr138 PMC3300837

[8] Hu M, Yao J, Carroll DK, Weremowicz S, Chen H, Carrasco D, et al Regulation of in situ to invasive breast carcinoma transition. Cancer Cell. 2008;13: 394–406. doi: 10.1016/j.ccr.2008.03.007 18455123 10.1016/j.ccr.2008.03.007 PMC3705908

[9] Kojima Y, Acar A, Eaton EN, Mellody KT, Scheel C, Ben-Porath I, et al Autocrine TGF-beta and stromal cell-derived factor-1 (SDF-1) signaling drives the evolution of tumor-promoting mammary stromal myofibroblasts. Proc Natl Acad Sci U S A. 2010;107: 20009–20014. doi: 10.1073/pnas.1013805107 21041659 10.1073/pnas.1013805107 PMC2993333

[10] Erez N, Truitt M, Olson P, Arron ST, Hanahan D. Cancer-Associated Fibroblasts Are Activated in Incipient Neoplasia to Orchestrate Tumor-Promoting Inflammation in an NF-kappaB-Dependent Manner. Cancer Cell. 2010;17: 135–147. doi: 10.1016/j.ccr.2009.12.041 20138012 10.1016/j.ccr.2009.12.041

[11] Yang G, Rosen DG, Zhang Z, Bast RC Jr., Mills GB, Colacino JA, et al The chemokine growth-regulated oncogene 1 (Gro-1) links RAS signaling to the senescence of stromal fibroblasts and ovarian tumorigenesis. Proc Natl Acad Sci U S A. 2006;103: 16472–16477. doi: 10.1073/pnas.0605752103 17060621 10.1073/pnas.0605752103 PMC1637606

[12] Ren C, Cheng X, Lu B, Yang G. Activation of interleukin-6/signal transducer and activator of transcription 3 by human papillomavirus early proteins 6 induces fibroblast senescence to promote cervical tumourigenesis through autocrine and paracrine pathways in tumour microenvironment. Eur J Cancer. 2013;49: 3889–3899. doi: 10.1016/j.ejca.2013.07.140 23953057 10.1016/j.ejca.2013.07.140

[13] Dean JP, Nelson PS. Profiling influences of senescent and aged fibroblasts on prostate carcinogenesis. Br J Cancer. 2008;98: 245–249. doi: 10.1038/sj.bjc.6604087 18182995 10.1038/sj.bjc.6604087 PMC2361445

[14] Krtolica A, Parrinello S, Lockett S, Desprez PY, Campisi J. Senescent fibroblasts promote epithelial cell growth and tumorigenesis: a link between cancer and aging. Proc Natl Acad Sci U S A. 2001;98: 12072–12077. doi: 10.1073/pnas.211053698 11593017 10.1073/pnas.211053698 PMC59769

[15] Parrinello S, Coppe JP, Krtolica A, Campisi J. Stromal-epithelial interactions in aging and cancer: senescent fibroblasts alter epithelial cell differentiation. J Cell Sci. 2005;118: 485–496. doi: 10.1242/jcs.01635 15657080 10.1242/jcs.01635 PMC4939801

[16] Liu J, Xu K, Chase M, Ji Y, Logan JK, Buchsbaum RJ. Tiam1-regulated osteopontin in senescent fibroblasts contributes to the migration and invasion of associated epithelial cells. J Cell Sci. 2012;125: 376–386. doi: 10.1242/jcs.089466 22302986 10.1242/jcs.089466 PMC3283874

[17] Illeperuma RP, Kim DK, Park YJ, Son HK, Kim JY, Kim J, et al Areca nut exposure increases secretion of tumor-promoting cytokines in gingival fibroblasts that trigger DNA damage in oral keratinocytes. Int J Cancer. 2015;137: 2545–2557. doi: 10.1002/ijc.29636 26076896 10.1002/ijc.29636 PMC4744697

[18] Lee EJ, Kim J, Lee SA, Kim EJ, Chun YC, Ryu MH, et al Characterization of newly established oral cancer cell lines derived from six squamous cell carcinoma and two mucoepidermoid carcinoma cells. Exp Mol Med. 2005;37: 379–390. doi: 10.1038/emm.2005.48 16264262 10.1038/emm.2005.48

[19] Orimo A, Weinberg RA. Stromal fibroblasts in cancer: a novel tumor-promoting cell type. Cell Cycle. 2006;5: 1597–1601. doi: 10.4161/cc.5.15.3112 16880743 10.4161/cc.5.15.3112

[20] Zhou B, Chen WL, Wang YY, Lin ZY, Zhang DM, Fan S, et al A role for cancer-associated fibroblasts in inducing the epithelial-to-mesenchymal transition in human tongue squamous cell carcinoma. J Oral Pathol Med. 2014;43: 585–592. doi: 10.1111/jop.12172 24645915 10.1111/jop.12172

[21] Chaudhri VK, Salzler GG, Dick SA, Buckman MS, Sordella R, Karoly ED, et al Metabolic alterations in lung cancer-associated fibroblasts correlated with increased glycolytic metabolism of the tumor. Mol Cancer Res. 2013;11: 579–592. doi: 10.1158/1541-7786.MCR-12-0437-T 23475953 10.1158/1541-7786.MCR-12-0437-T PMC3686965

[22] Taddei ML, Cavallini L, Comito G, Giannoni E, Folini M, Marini A, et al Senescent stroma promotes prostate cancer progression: the role of miR-210. Mol Oncol. 2014;8: 1729–1746. doi: 10.1016/j.molonc.2014.07.009 25091736 10.1016/j.molonc.2014.07.009 PMC5528604

[23] Hassona Y, Cirillo N, Lim KP, Herman A, Mellone M, Thomas GJ, et al Progression of genotype-specific oral cancer leads to senescence of cancer-associated fibroblasts and is mediated by oxidative stress and TGF-beta. Carcinogenesis. 2013;34: 1286–1295. doi: 10.1093/carcin/bgt035 23358854 10.1093/carcin/bgt035

[24] Rojas A, Liu G, Coleman I, Nelson PS, Zhang M, Dash R, et al IL-6 promotes prostate tumorigenesis and progression through autocrine cross-activation of IGF-IR. Oncogene. 2011;30: 2345–2355. doi: 10.1038/onc.2010.605 21258401 10.1038/onc.2010.605 PMC3112005

[25] Son HK, Park I, Kim JY, Kim do K, Illeperuma RP, Bae JY, et al A distinct role for interleukin-6 as a major mediator of cellular adjustment to an altered culture condition. J Cell Biochem. 2015;116: 2552–2562. doi: 10.1002/jcb.25200 25939389 10.1002/jcb.25200 PMC4832257

[26] Opdenakker G, Van Damme J. The countercurrent principle in invasion and metastasis of cancer cells. Recent insights on the roles of chemokines. Int J Dev Biol. 2004;48: 519–527. doi: 10.1387/ijdb.041796go 15349826 10.1387/ijdb.041796go

[27] Bandapalli OR, Ehrmann F, Ehemann V, Gaida M, Macher-Goeppinger S, Wente M, et al Down-regulation of CXCL1 inhibits tumor growth in colorectal liver metastasis. Cytokine. 2012;57: 46–53. doi: 10.1016/j.cyto.2011.10.019 22129625 10.1016/j.cyto.2011.10.019

[28] Chien Y, Scuoppo C, Wang X, Fang X, Balgley B, Bolden JE, et al Control of the senescence-associated secretory phenotype by NF-kappaB promotes senescence and enhances chemosensitivity. Genes Dev. 2011;25: 2125–2136. doi: 10.1101/gad.17276711 21979375 10.1101/gad.17276711 PMC3205583

[29] Kolar M, Szabo P, Dvorankova B, Lacina L, Gabius HJ, Strnad H, et al Upregulation of IL-6, IL-8 and CXCL-1 production in dermal fibroblasts by normal/malignant epithelial cells in vitro: Immunohistochemical and transcriptomic analyses. Biol Cell. 2012;104: 738–751. doi: 10.1111/boc.201200018 23043537 10.1111/boc.201200018

[30] Zhang H, Yue J, Jiang Z, Zhou R, Xie R, Xu Y, et al CAF-secreted CXCL1 conferred radioresistance by regulating DNA damage response in a ROS-dependent manner in esophageal squamous cell carcinoma. Cell Death Dis. 2017;8: e2790 doi: 10.1038/cddis.2017.180 28518141 10.1038/cddis.2017.180 PMC5520705

