# Supplementary figures and images for: CXCL1 induces senescence of cancer-associated fibroblasts via autocrine loops in oral squamous cell carcinoma
Source: PLoS One. 2018 Jan 23;13(1):e0188847. doi: 10.1371/journal.pone.0188847 (PMC5779641; doi:10.1371/journal.pone.0188847)

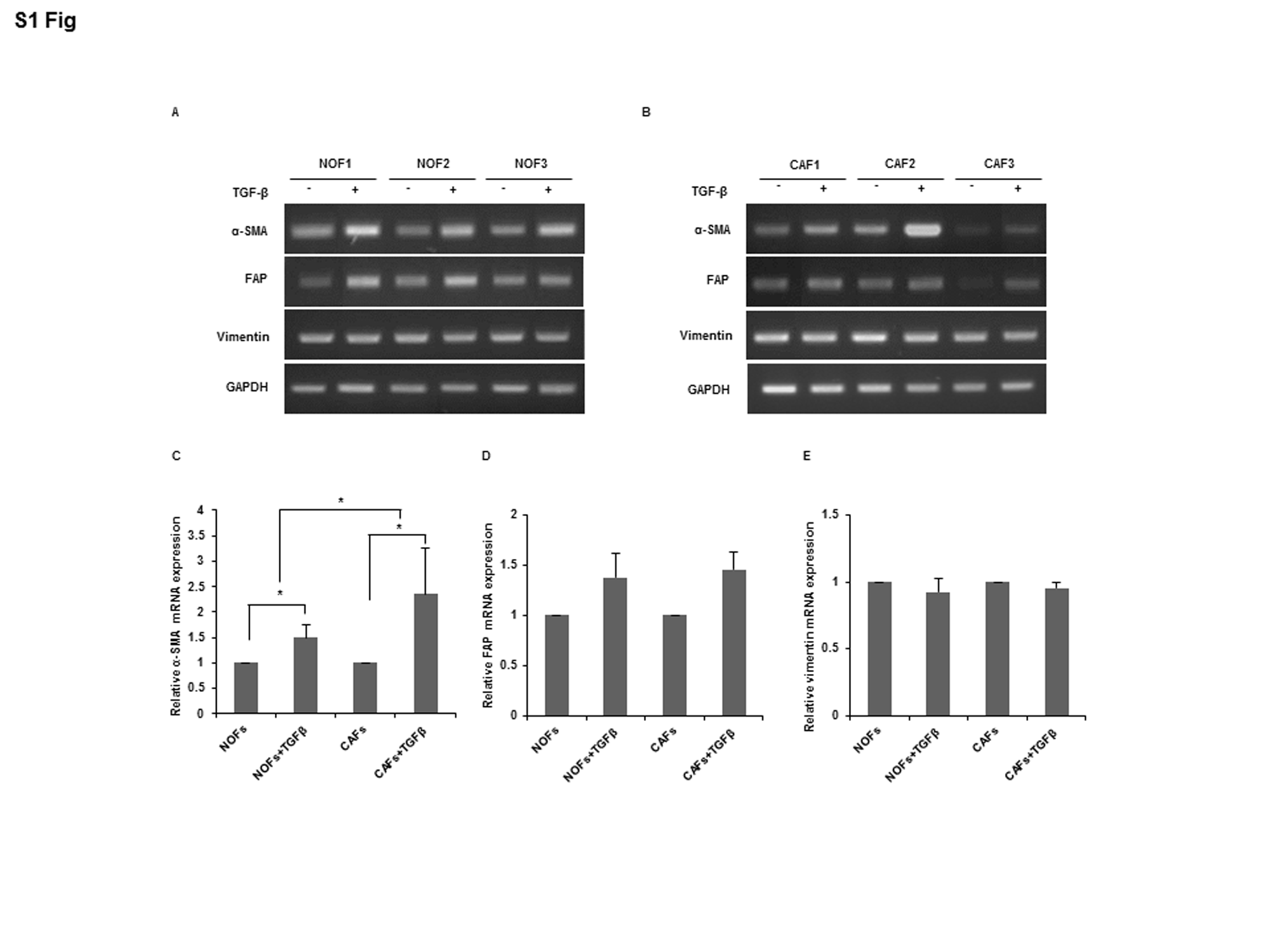

Supplement: S1 Fig — (A,B) α-SMA, FAP, and vimentin mRNA expression according to TGF-β treatment in NOFs (A) and CAFs (B). The graphs are presented as the mean value ± SD in triplicates. (C, D, E) Densitometric analysis of mRNA for α-SMA (C), FAP (D), and vimentin (E) were carried out with a loading control of GAPDH expression. The results are presented as the mean value ± SD in triplicates and were analyzed by the Mann-Whitney U test (*p < 0.05). (TIF) [file pone.0188847.s001.tif]

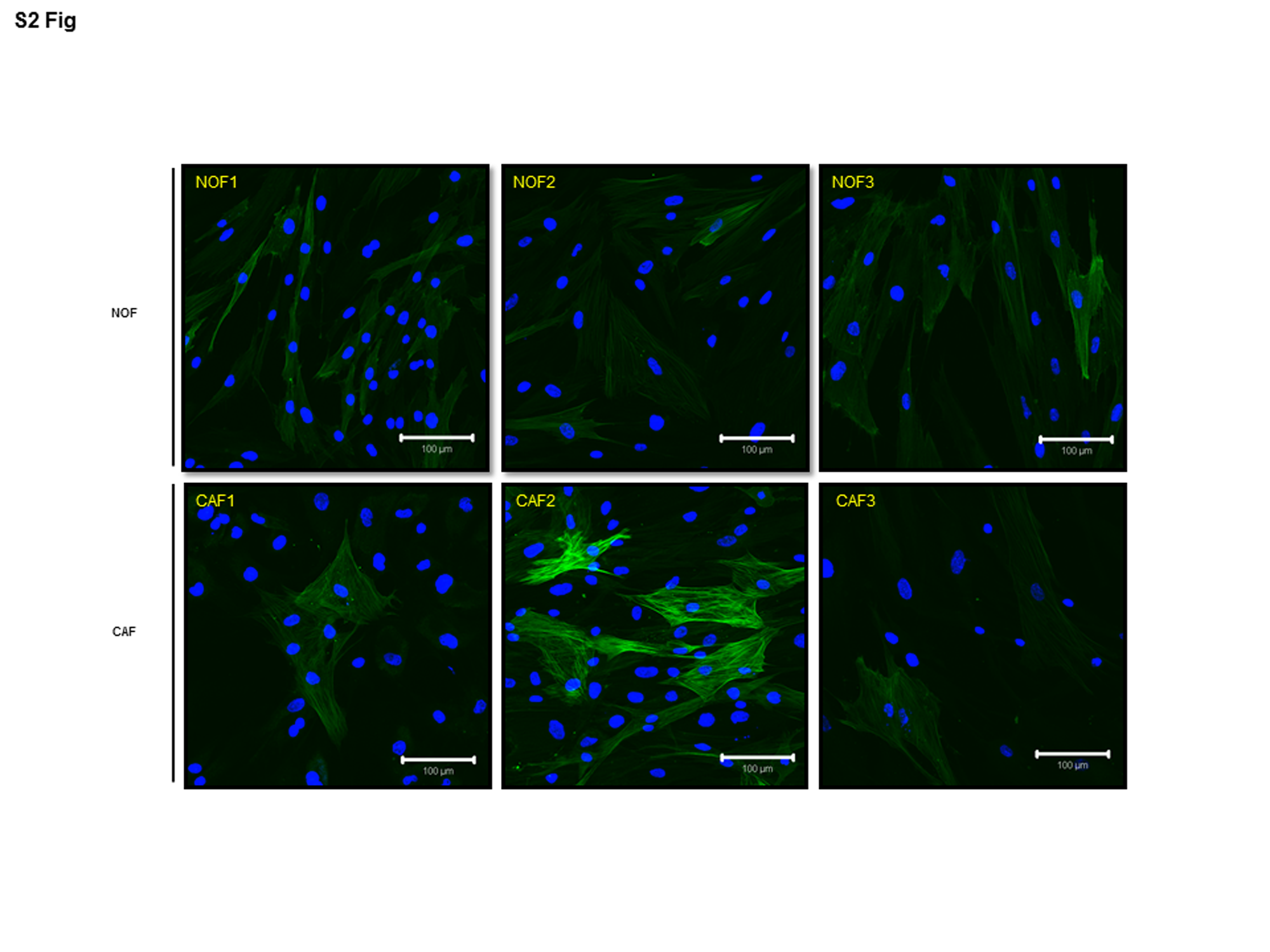

Supplement: S2 Fig — Images are shown the basal levels of α-SMA in NOFs(upper) and CAFs(lower). Merged staining was shown (DAPI(blue), α-SMA(Green)). (magnification: X200, scale bar: 100μm). (TIF) [file pone.0188847.s002.tif]

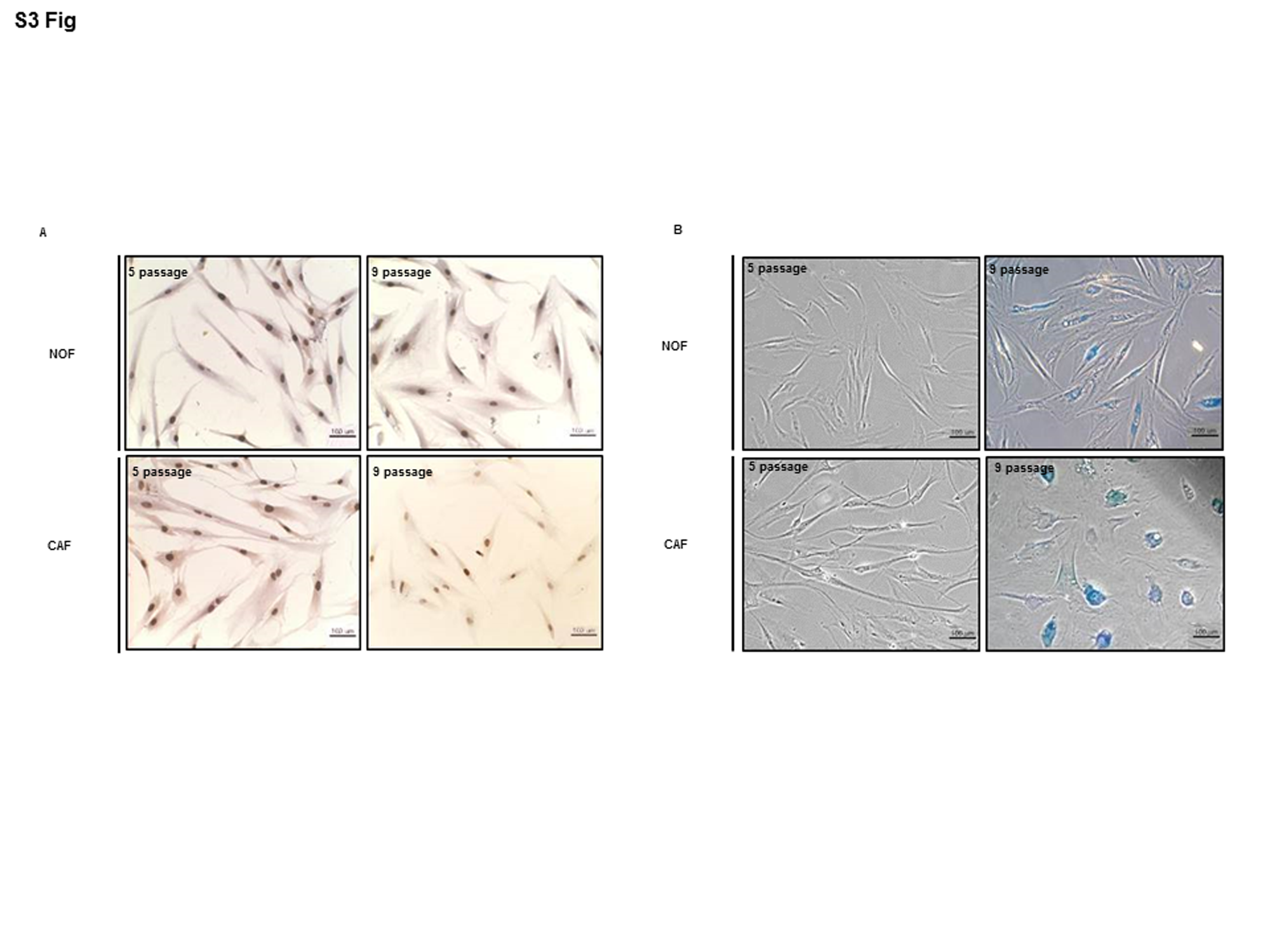

Supplement: S3 Fig — (A) The positive cells for PCNA show the brown to black colored nuclei. (B) The positive cells for SA-β-Gal show the blue colored cytoplasm and nuclei. (magnification: X 200, scale bar: 100μm). (TIF) [file pone.0188847.s003.tif]

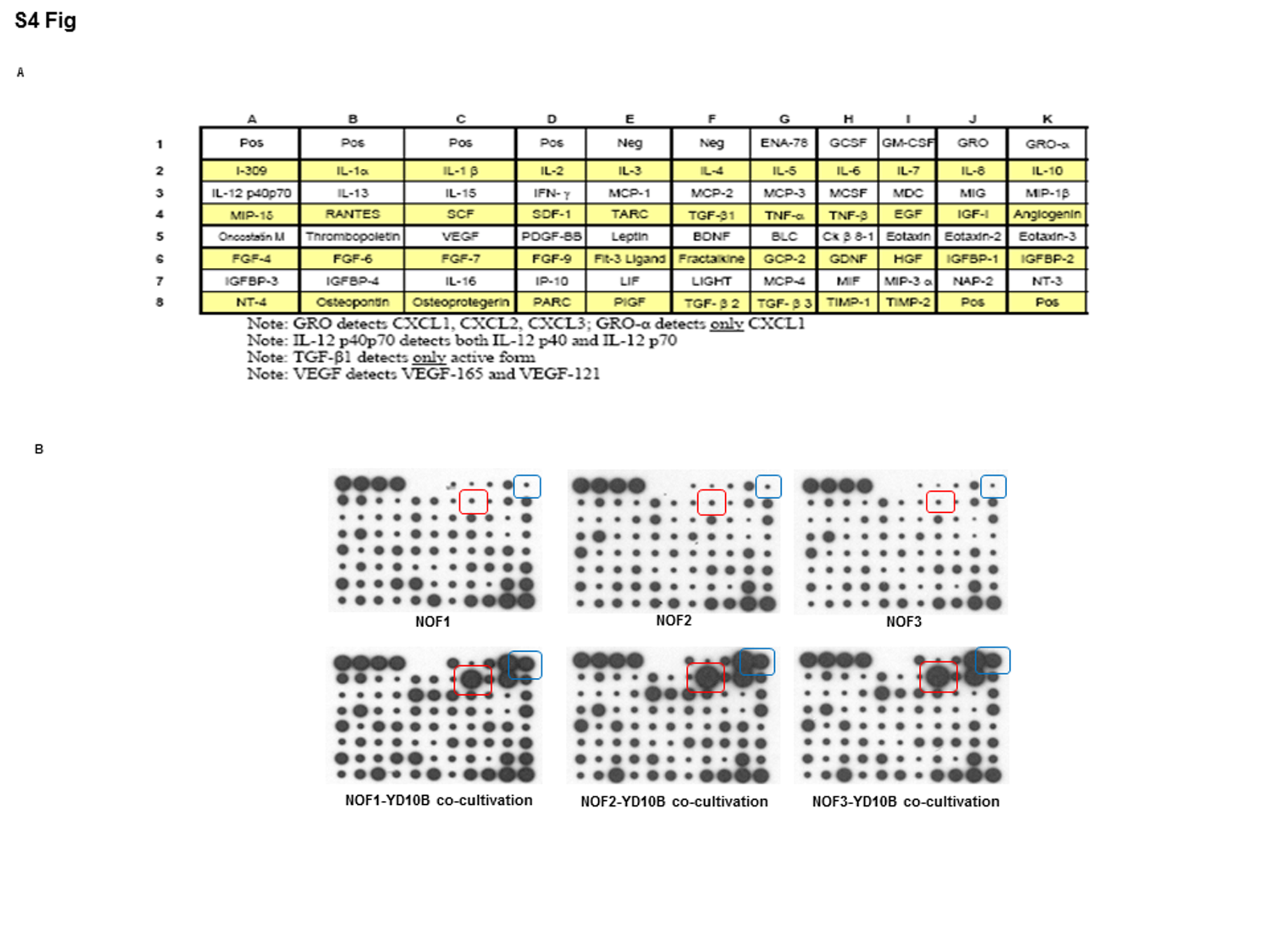

Supplement: S4 Fig — (A) The RayBio Human Cytokine Antibody Array Map. A total of 80 antibodies against cytokines, negative control (Neg), and positive control (Pos) were included in the array (B) Representative pictures of cytokine antibody array in mono-culture NOFs and co-cultured NOFs with YD10B OSCC cells. IL-6 (identified by red empty squares) and CXCL1 (identified by blue empty squares) are the highest secretion in conditioned medium from co-culture with NOFs and YD10B OSCC cells compared to mono-cultured cells. (TIF) [file pone.0188847.s004.tif]

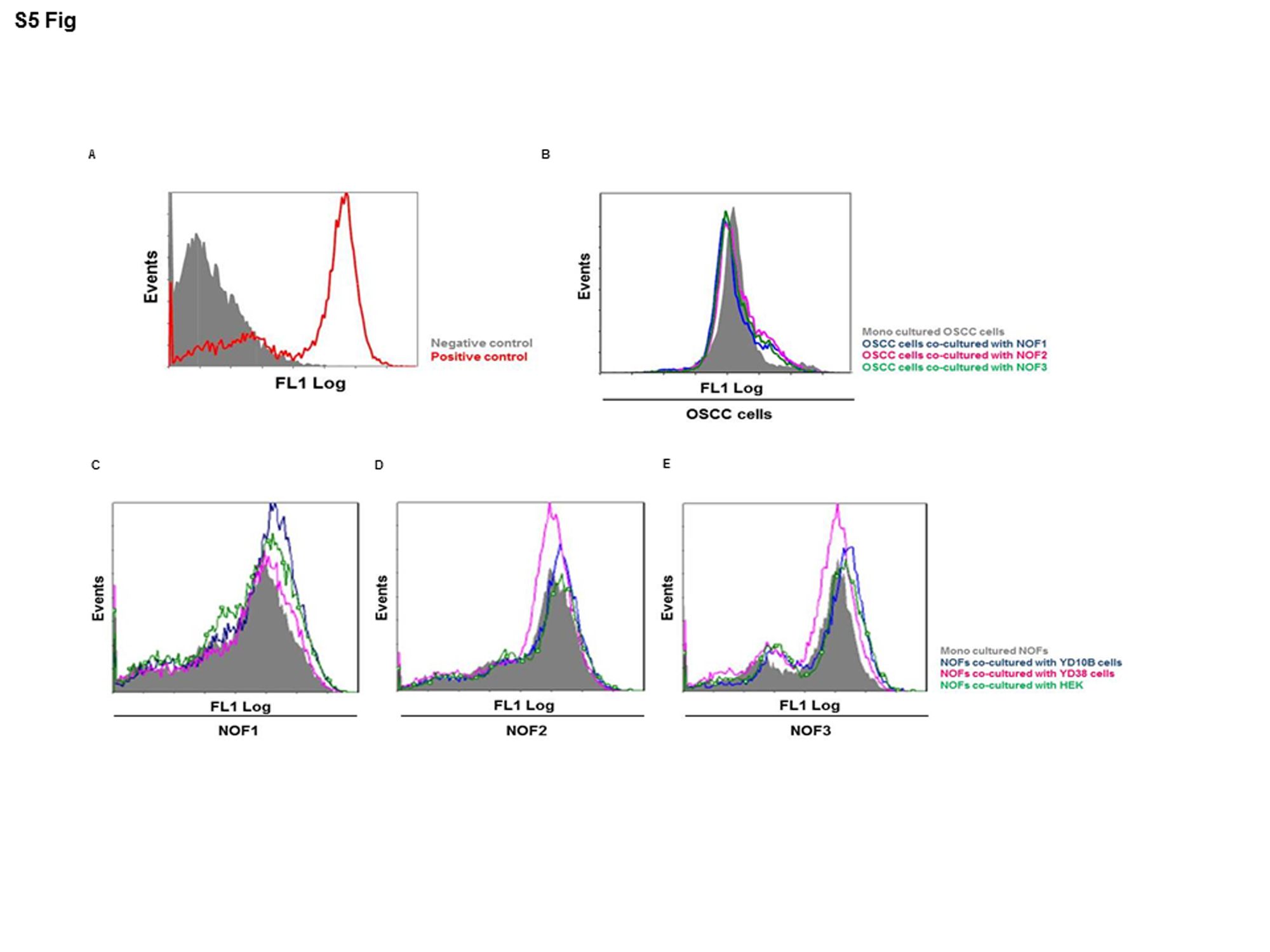

Supplement: S5 Fig — Flow cytometry analysis of positive cell stained H2DCFDA dye for detection of ROS generation in mono-culture and co-culture condition. (A) Negative (H2DCFD-non treatment) and positive (10 μM H2O2 treatment) control (B) mono-cultured OSCC cells and OSCC cells co-cultured with NOFs (C, D, E) mono-cultured NOFs and NOFs co-cultured with OSCC cells. (TIF) [file pone.0188847.s005.tif]

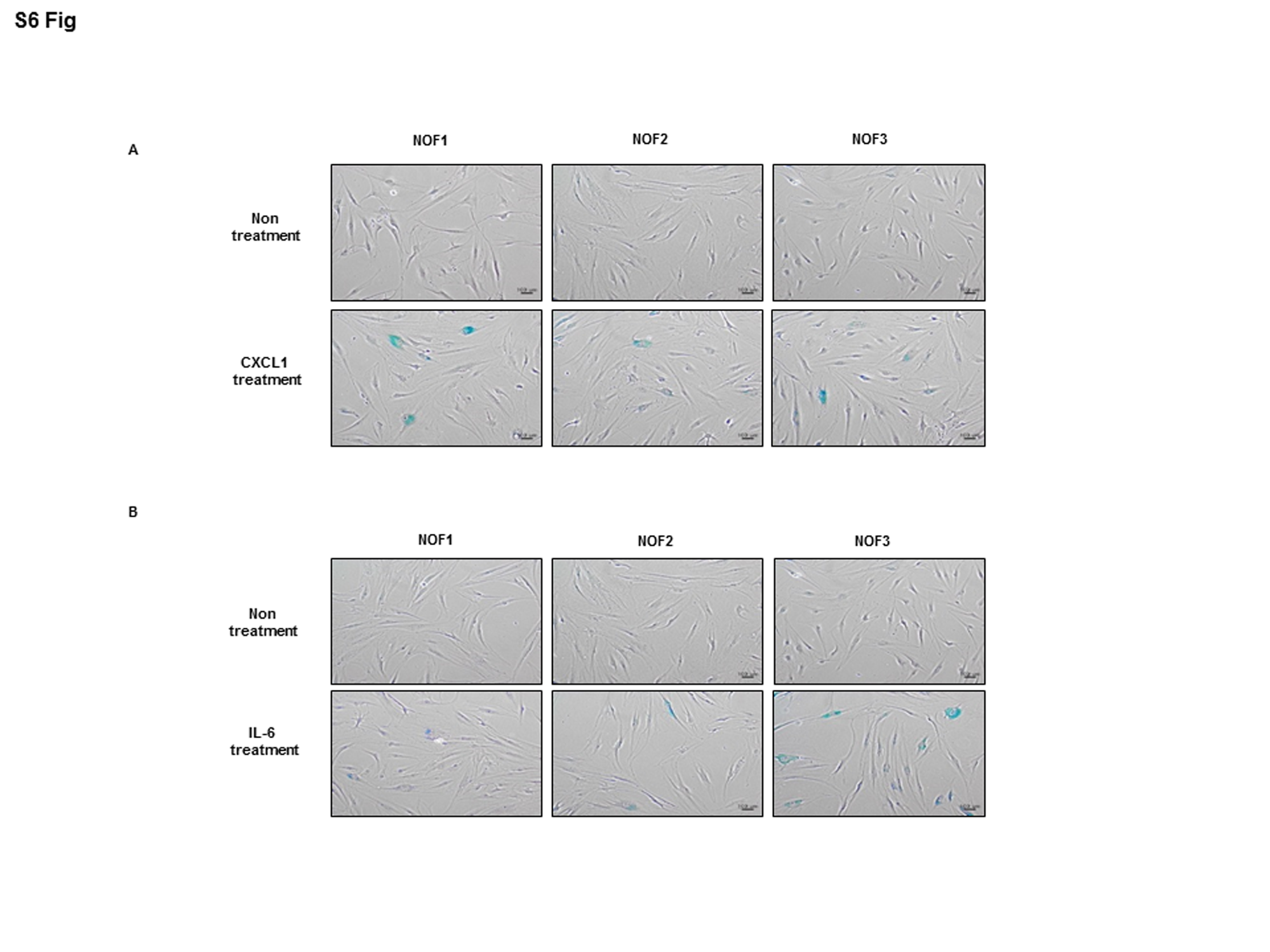

Supplement: S6 Fig — The treatment with CXCL1 (A) and IL-6 recombinant protein (B) (magnification: 200X, Scale bar: 100 μm). (TIF) [file pone.0188847.s006.tif]

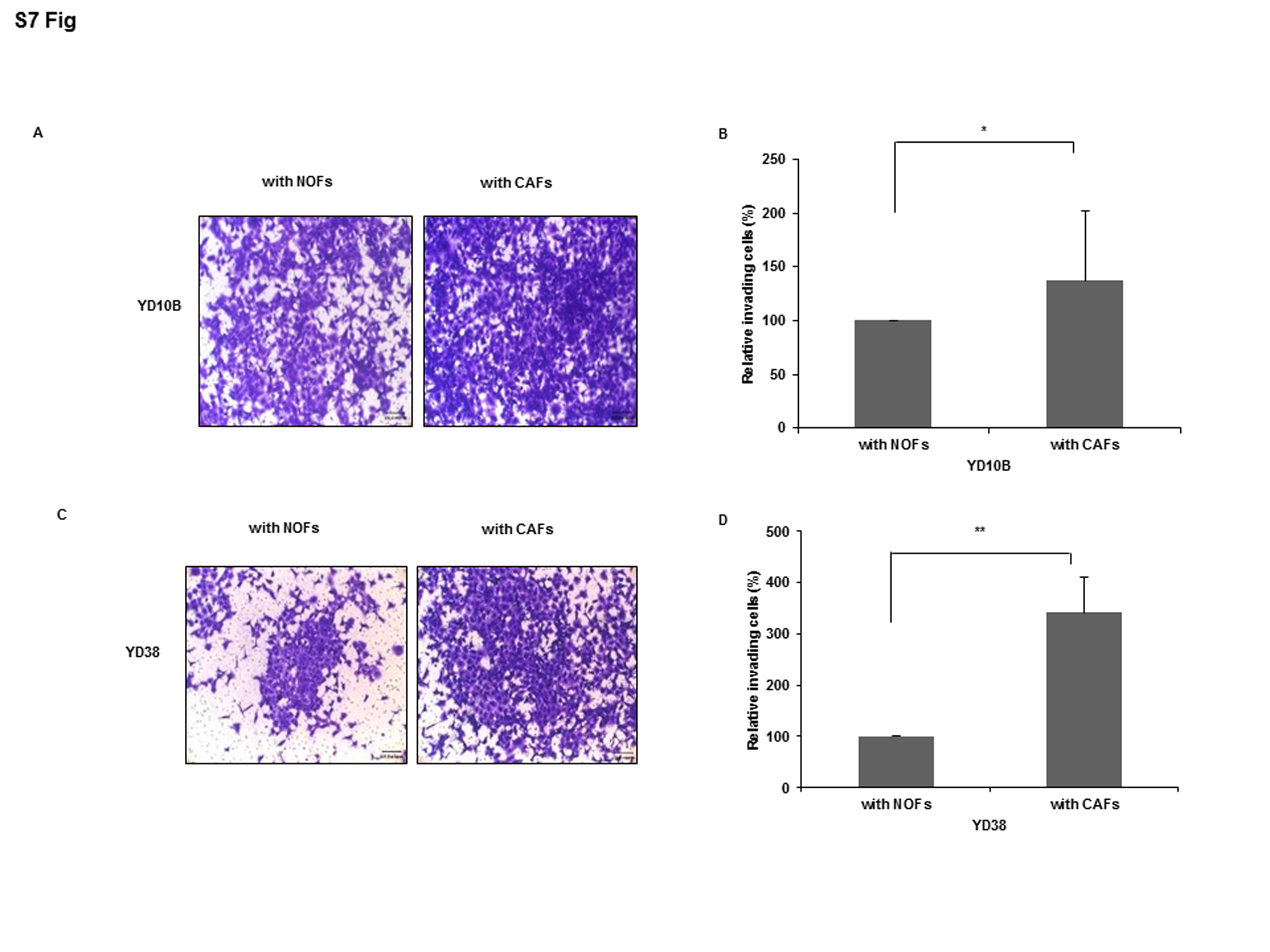

Supplement: S7 Fig — YD10B (A,B) or YD38 (C,D) cells in serum-free media were placed in the upper well of a 24-transwell plate with collagen-coated filters (8 μm pore). NOFs or CAFs was added into the lower well to induce invasion. The invasive cells were counted after 48 h by light microscopy. (A,C) Representative microscopic pictures of invading YD10B or YD38 OSCC cells (magnification: 100X, scale bar: 100 μm). (B,D) The number of invasive cells was normalized by dividing by the number of total cells and presented as the percentage of invasion. The results are presented as the mean value ± SD in triplicates and were analyzed by the Mann-Whitney U test (**p < 0.01, *p < 0.05). (TIF) [file pone.0188847.s007.tif]
