# Supplementary material for: CXCL1 induces senescence of cancer-associated fibroblasts via autocrine loops in oral squamous cell carcinoma
Source: PLoS One. 2018 Jan 23;13(1):e0188847. doi: 10.1371/journal.pone.0188847 (PMC5779641; doi:10.1371/journal.pone.0188847)
Supplement: S1 Table — The preliminary tables indicated to check the concentration of each cytokine secreted in mono-cultured or co-cultured NOFs with OSCC cells for 48 h. For following experiments, the optimal concentration of recombinant human IL-6 (7 ng/ml; Top table) and CXCL1 (5 ng/ml; Middle table) were applied in NOFs for 48 h. The optimal concentration of CXCL1 neutralizing antibody (20 μg/ml; bottom table) was determined as the most effective reduction of CXCL1 secretion. (DOCX) [file pone.0188847.s008.docx]

**S1 Table.** The preliminary study for optimal concentration of IL-6, CXCL1 and CXCL1 neutralizing antibody

|  | **IL-6 secretion**  **(pg/ml)** |
| --- | --- |
| **NOFs** | 37.4009 ± 5.00391 |
| **Co-culture with NOFs and YD10B** | 7419.21 ± 759.911 |

|  | **CXCL1 secretion**  **(pg/ml)** |
| --- | --- |
| **NOFs** | 200.476 ± 253.388 |
| **Co-culture with NOFs and YD10B** | 5121.44 ± 280.384 |

| **CXCL1 neutralizing Ab**  (**ug)** | **CXCL1 secretion reduction**  **(pg/ml) (%)** |
| --- | --- |
| **YD10B-NOF1** | 4247.50 0 |
| **5ug** | 701.66 83.5 |
| **10ug** | 622.50 85.5 |
| **20ug** | 405.83 90.5 |
