# Supplementary material for: CXCL1 induces senescence of cancer-associated fibroblasts via autocrine loops in oral squamous cell carcinoma
Source: PLoS One. 2018 Jan 23;13(1):e0188847. doi: 10.1371/journal.pone.0188847 (PMC5779641; doi:10.1371/journal.pone.0188847)
Supplement: S2 Table — Images of randomly selected 5 microscopic fields (magnification: X200) were acquired per sample (Olympus, Tokyo, Japan). The average (%) was indicated with standard deviation. (DOCX) [file pone.0188847.s009.docx]

**S2 Table.** The percentage of PCNA-positive cells in NOFs and CAFs according to passages

|  |  | **5^th^ passage** | **6^th^ passage** | **7^th^ passage** | **8^th^ passage** | **9^th^ passage** |
| --- | --- | --- | --- | --- | --- | --- |
| **Average**  **(%)** | **NOFs** | 64.14 ± 4.66 | 56.50 ± 6.07 | 50.12 ± 5.06 | 34.56 ± 8.54 | 28.18 ± 11.06 |
|  | **CAFs** | 56.93 ± 8.15 | 53.43 ± 6.06 | 40.37 ± 18.79 | 21.13 ± 15.14 | 18.73 ± 13.33 |
