# Supplementary material for: CXCL1 induces senescence of cancer-associated fibroblasts via autocrine loops in oral squamous cell carcinoma
Source: PLoS One. 2018 Jan 23;13(1):e0188847. doi: 10.1371/journal.pone.0188847 (PMC5779641; doi:10.1371/journal.pone.0188847)
Supplement: S3 Table — Images of randomly selected 5 microscopic fields (magnification: X200) were acquired per sample (Olympus, Tokyo, Japan). The average (%) was indicated with standard deviation. (DOCX) [file pone.0188847.s010.docx]

**S3 Table.** The percentage of SA-β-Gal-positive cells in NOFs and CAFs according to passages

|  |  | **5^th^ passage** | **6^th^ passage** | **7^th^ passage** | **8^th^ passage** | **9^th^ passage** |
| --- | --- | --- | --- | --- | --- | --- |
| **Average**  **(%)** | **NOFs** | 12.85 ± 2.83 | 49.24 ± 1.31 | 59.17 ± 7.55 | 56.20 ± 12.16 | 63.43 ± 10.83 |
|  | **CAFs** | 20.69 ± 7.27 | 22.58 ± 6.30 | 62.12 ± 10.32 | 73.50 ± 18.68 | 72.20 ± 18.92 |
