# Supplementary material for: CXCL1 induces senescence of cancer-associated fibroblasts via autocrine loops in oral squamous cell carcinoma
Source: PLoS One. 2018 Jan 23;13(1):e0188847. doi: 10.1371/journal.pone.0188847 (PMC5779641; doi:10.1371/journal.pone.0188847)
Supplement: S1 Materials and Methods — (DOCX) [file pone.0188847.s011.docx]

**Supporting information – S1 Materials and Methods**

**Cell culture**

In brief, connective tissue was cut into small pieces and the tissues were placed in the culture medium composed of Dulbecco’s modified Eagles medium (Gibco BRL, NY, USA) and F-12 Ham (Ham’s F12; Gibco BRL, NY, USA) mixed in a 3:1 ratio, and supplemented with 10% fetal bovine serum, 1% penicillin/streptomycin, 0.01µg/ml cholera toxin, 0.04 µg/ml hydrocortisone, 0.5 µg/ml insulin, 0.5 ug/ml apo-transferrin, and 0.2 µg/ml 3´-5-triodo-1-thyroine (Sigma, MO, USA). When the cells covered the bottom of the culture dish, cells were detached using 0.05% trypsin-EDTA solution (Gibco BRL, NY, USA). NOFs and CAFs were then maintained in culture medium composed of Dulbecco’s modified Eagles medium (Gibco BRL, NY, USA) and F-12 Ham (Ham’s F12; Gibco BRL, NY, USA) mixed in a 3:1 ratio, and supplemented with 10% fetal bovine serum and 1% penicillin/streptomycin. HEK were maintained in keratinocyte growth media (KGM; Lonza, Walkersville, MD, USA) with supplementary bullet kit (Lonza, Walkersville, MD, USA). YD-10B and YD-38 OSCC cells were maintained in culture medium composed of Dulbecco’s modified Eagles medium (Gibco BRL, NY, USA) and F-12 Ham (Ham’s F12; Gibco BRL, NY, USA) mixed in a 3:1 ratio, and supplemented with 10% fetal bovine serum, 1% penicillin/streptomycin, 0.01 µg/ml cholera toxin, 0.04 µg/ml hydrocortisone, 0.5 µg/ml insulin, 0.5 ug/ml apo-transferrin, and 0.2 µg/ml 3´-5-triodo-1-thyroine (Sigma, MO, USA). All cells were cultured at 37 °C in an incubator that contained 5% CO_2_.
