# Supplementary material for: CXCL1 induces senescence of cancer-associated fibroblasts via autocrine loops in oral squamous cell carcinoma
Source: PLoS One. 2018 Jan 23;13(1):e0188847. doi: 10.1371/journal.pone.0188847 (PMC5779641; doi:10.1371/journal.pone.0188847)
Supplement: S2 Materials and Methods — (DOCX) [file pone.0188847.s012.docx]

**Supporting information – S2 Materials and Methods**

**Immunofluorescence**

NOFs and CAFs (5 × 10^4^) seeded onto Lab-Tek™ chamber slide (Nalge Nunc, Roskilde, Denmark) and then stabilized for 24 h before staining. The cells were washed with PBS and then fixed with 4% formaldehyde solution in PBS. After fixation, cells were applied with bovine serum albumin for 30 min, and stained with the FITC-conjugated anti-α-SMA (1:200, Sigma, MO, USA) for overnight at – 4ºC. Next, cells were washed with PBS to remove unbound α-SMA and then applied to stain the nucleus with 10 μg/ml diamidino-2-phenylindole(DAPI)(Sigma, MO, USA) for 30 min. Lastly, cells cover-slipped with Dako fluorescent mounting solution (Dako, Glostrup, Denmark), visualized and photographed using confocal microscopy (LSM 700, Carl Zeiss, Germany).
