# Supplementary material for: CXCL1 induces senescence of cancer-associated fibroblasts via autocrine loops in oral squamous cell carcinoma
Source: PLoS One. 2018 Jan 23;13(1):e0188847. doi: 10.1371/journal.pone.0188847 (PMC5779641; doi:10.1371/journal.pone.0188847)
Supplement: S3 Materials and Methods — (DOCX) [file pone.0188847.s013.docx]

**Supporting information – S3 Materials and Methods**

**Preparation of conditioned medium**

In mono-culture, NOFs or OSCC cells (2.5 × 10^5^) were seeded in 6-well plates. In co-culture, 2.5 × 10^5^ OSCC cells and 2.5 × 10^5^ NOFs were seeded in the upper chamber and lower chamber, respectively, of 6-transwell plates containing collagen-coated 0.4-µm pore transmembrane filters (Corning-costar, Lowell, MA, USA). Each culture was maintained with 3 ml serum-free DMEM: Hams-F12 (3:1) for 48 h. The supernatant was centrifuged, collected and stored at -80 °C.
